# Perioperative glycaemic control for preterm infant with transient neonatal hyperglycaemia and gastroschisis

**DOI:** 10.1186/s13104-016-1957-y

**Published:** 2016-03-03

**Authors:** Sirirat Rattana-arpa, Saowaphak Lapmahapaisan, Arunotai Siriussawakul

**Affiliations:** Department of Anesthesiology, Faculty of Medicine, Siriraj Hospital, Mahidol University, Bangkok, 10700 Thailand

**Keywords:** Abdominal wall defect, High blood sugar, Prematurity

## Abstract

**Background:**

Neonatal hyperglycaemia is a rare metabolic disorder. There are no reports of an association between neonatal hyperglycaemia and gastroschisis.

**Case presentation:**

This report presents preoperative and intraoperative management of blood sugar in a low birth weight Thai preterm neonate with gastroschisis and a diagnosis of neonatal hyperglycaemia. The patient underwent an emergency, multi-staged, surgical repair under general anaesthesia.

**Conclusion:**

Anaesthesiologists should be aware of possible perioperative dysglycaemic conditions in these patients. Proper timing of surgery and appropriate preanaesthetic preparation are necessary to reduce the morbidity and mortality related to hyperglycaemia and gastroschisis.

**Consent:**

The patient’s guardian has given consent for the case report to be published.

## Background

Dysglycaemia frequently occurs in very low birth weight preterm infants. Deficiency of glycogen stores at birth and defective counter-regulatory hormone responses are common in prematurity, making hypoglycaemia a more frequent occurrence than hyperglycaemia [1]. The incidence of neonatal hyperglycaemia has not been clearly reported because there is no consensus on its definition. Most reports have defined neonatal hyperglycaemia as blood glucose concentrations >7 mmol/L (>126 mg/dL) for term infants, and >8.3 mmol/L (>150 mg/dL) for preterm infants [1, 2].

Gastroschisis is a congenital abdominal wall defect which occurs in 1 in 15,000 live births. This condition rarely has associated abnormalities [3], and there is no previous report of gastroschisis with neonatal hyperglycaemia. Generally, infants with gastroschisis require emergency surgical correction in order to prevent further fluid loss and infection [3]. Nevertheless, the timing of the surgical correction of the gastroschisis in this particular case was somewhat controversial. This report demonstrates the challenge of decision-making by a multidisciplinary team that analysed the risks and benefits of promptly going to surgery versus waiting to optimize the patient’s condition.

## Case report

Written, informed consent was obtained from the patient’s guardian for the publication of this case report.

A Thai male baby was born by elective caesarean section at 34 weeks of gestation by a 19-year-old, primigravida Thai female at a private hospital. The mother did not have any major diseases, and there was no family history of diabetes mellitus. No evidence of complications of pregnancy was found during the antenatal period. Gastroschisis was detected by prenatal ultrasonography during routine screening. Bowel loops were seen herniating into the amniotic cavity, and they were floating without any covering membrane. Dexamethasone (5 mg intramuscularly, with 4 doses 12-hourly) was then administered before pregnancy termination. The baby boy’s birth weight was 1590 g, appropriate for his gestational age. The Apgar scores at the 1st and the 5th min were 9 and 10, respectively. The baby was active, and no associated anomalies were detected.

The baby was shifted to the Neonatal Intensive Care Unit (NICU). The intestinal loops were enclosed in a sterile plastic sheet and placed in midline. The blood sugar level was checked immediately, with a result of 114 mg/dL (6.3 mmol/L). Six milliliters per hour (ml/h) of 10 % Dextrose and one-fifth normal saline (10 %D/N/5) were administered with a glucose infusion rate, or GIR, of 6.28 mg/kg/min. A complete blood count and serum electrolyte test were performed, but the results were normal.Subsequently, the baby was transferred to a university tertiary hospital for repair of the gastroschisis. Upon his arrival, the blood sugar level was checked in his 4th hour of life by point-of-care testing (POCT, the Lifescan SureStep Flexx Meter, Lifescan, Inc., United States). The result showed “high”, which was equivalent to a glucose concentration >600 mg/dL in the pertinent plasma. An analysis of the plasma glucose by a laboratory test was not able to be performed because of a difficult venipuncture. However, laboratory tests did show negative findings of urine ketone and urine sugar. The decrement of GIR was adjusted in order to decrease the blood glucose levels. The intravenous fluid was changed to 5 %D/N/5 to keep the GIR at 4–8 mg/kg/min. The POCT was repeated hourly, but the results still revealed “high” even though the GIR was further reduced to 1.65 mg/kg/min at the 6th hour of life. Gastroschisis is considered to be a surgical emergency requiring the closure or coverage of the abdominal contents after birth. A multi-disciplinary team, including paediatric surgeons, anaesthesiologists and neonatologists, were enrolled to discuss the risks and the benefits of delaying surgery. They were concerned about evaporation and heat loss. Stress and infection would also worsen the hyperglycaemic condition. The team finally decided to send the patient to the operative room because of the large abdominal wall defect that needed a sterile site and some special equipment. Meanwhile, fluid resuscitation was performed and POCT was rechecked.

In the operating room, standard monitoring was performed. It included pre- and post-ductal oximetry, an electrocardiogram, non-invasive blood pressure, capnography, temperature, POCT and urine output. A general anaesthesia utilising an endotracheal tube was inducted by administering thiopental and intubating with succinylcholine. The anaesthesia was maintained by using fentanyl, atracurium, isoflurane, air and oxygen. The intraoperative POCT sustained high during the 1st hour of the surgery. The intravenous regular insulin infusion was started at 0.06 units/kg/h, and the glucose infusion rate was reduced to 1.57 mg/kg/min in the 2nd hour. One hour after initiation of the regular insulin, the POCT reading of the baby’s blood sugar level was still immeasurably high. A constant, intravenous glucose infusion rate was maintained. Then, the regular insulin infusion dose was increased from 0.06 to 0.1 units/kg/h, and blood sugar level was checked.

The staged repair, utilising a protective silo placement, lasted for 3 h. The patient remained intubated and received a regular insulin infusion dose of 0.1 units/kg/h while being transferred to the neonatal surgical ICU. His blood sugar level had a tendency to decrease at the 10th hour of life. The first measurable result by POCT was 406 mg/dL, and the venous blood sugar confirmation at that time was 426 mg/dL. Then, 0.06 units/kg/h of intravenous regular insulin infusion was given for 4 h. The regular insulin was stopped at the 14th hour of life when the blood sugar result was 161 mg/dL. The blood sugar level, the insulin infusion and the intraoperative management are summarised at Table 1. Neither seizure nor convulsion was found during the perioperative period.

**Table 1:** Perioperative glucose management, point-of-care glucose results and venous blood glucose in the first 15 h

Age (h)	GIR (mg/kg/min)	Insulin (unit/kg/h)	POCT (mg/dL)	Venous blood test (mg/dL)
0	6.28	0	114	–
1	6.28	0	–	–
2	6.28	0	–	–
3	6.28	0	–	–
4	6.28	0	High	–
5	3.14	0	High	–
6	1.65	0	High	–
7	1.57	0.06	High	–
8	1.57	0.1	High	–
9	1.57	0.1	High	–
10	1.57	0.06	406	426
11	1.57	0.06	326	–
12	1.57	0.06	305	–
13	1.57	0.06	263	–
14	1.57	0	161	–
15	1.57	0	70	–

*GIR* glucose infusion rate, *POCT* point of care testing of blood sugar

The baby was scheduled for a fascial closure on his 3rd day of life. The surgery was uneventful, and he was discharged to home after 2 weeks of admission. His blood sugar level was in the normal range from his 14th hour of life until the hospital discharge, and he did not demonstrate any hyperglycaemic episodes. During the long-term follow-up at the 3rd, 8th, and 20th months of age, the child presented with normal growth and development without any signs of metabolic syndrome or neurological problems.

## Discussion

Gastroschisis is an emergency, surgical condition in preterm infants [4]. This case was unusual in that the patient presented with hyperglycaemia. The hyperglycaemic condition could have arisen from a multitude of factors, e.g., stress induced hyperglycaemia, infection and iatrogenic intravenous glucose loading. The stress response to the surgery and the existing hyperglycaemia may cause perioperative ketoacidosis or hyperosmolar syndrome. However, delaying surgery results in ongoing hypovolaemia and an increased risk of sepsis, which can worsen glucose homeostasis and metabolic disorder [3]. As a result, morbidity and mortality increase [2, 5]. A multi-disciplinary team, including surgeons, paediatricians and anaesthesiologists, should correct the underlying causes immediately. In general, stress correction is usually sufficient to improve the hyperglycaemic condition [6]. Important, significant factors for this patient were the timing of the surgery and strategies for perioperative glycaemic control.

This patient met the diagnostic definition of transient neonatal hyperglycaemia [1, 2, 7]. The first management-step for hyperglycaemic patients is to confirm the diagnosis and search for possible causes. The probable causes of hyperglycaemia in this case were a counter-regulatory response to stress, gastroschisis and immature organ function.Other causes of neonatal hyperglycaemia could be the combination of a decreased amount of insulin production, inadequate insulin response to perinatal stress, drugs (steroid, Beta-2 agonist, phenytoin, theophylline), genetic and the amount of glucose infusion [1, 2, 6, 8]. A maternal dexamethasone injection for foetal lung maturation may result in high blood sugar levels in the pregnant mothers [9]. No previous study had revealed the relationship between a maternal dexamethasone injection and the neonatal blood glucose levels. Genetic factors such as chromosomal abnormalities in the imprinted region of chromosome 6q24, or mutations in genes KCNJ11 (Kir6.2) or ABCC8 (SUR1), could be commonly found in patients with transient neonatal diabetes [7, 10].However, genetic testing of the patient was not done because the hyperglycaemia was transient.

Concerning the morbidity and mortality of this condition, e.g., osmotic diuresis, sepsis, and a longer hospital stay, most institutions recommend starting treatment [2, 5]. Treatment options could be doing nothing, reducing the amount of glucose given, treating the patient with insulin, or all of the above alternatives [1, 2, 8, 11]. Nevertheless, there is currently no scientifically-supported reference value and standard guideline for the management of a neonatal hyperglycaemic condition. A goal for the paediatric, intra-operative blood glucose range is still being debated.In the case of paediatric, cardiac-surgery patients, it has been suggested that maintaining a glucose level of less than 7.8 mmol/L (140 mg/dL) would reduce morbidity, mortality and improve patient outcomes [12]. Due to the persistently high blood sugar level of this infant, a regular insulin infusion was given to facilitate glucose intake after a period of reduced glucose intake [13]. Many institutions have created and developed clinical practice guidelines for regular insulin infusion therapy for neonates. The starting dose of the regular insulin varies from 0.02 to 0.1 units/kg/h. Subsequently, the regular insulin infusion rate should be adjusted hourly by 0.01–0.02 units/kg/h, according to the patients’ responses. Regular blood sugar monitoring should be done while administering the insulin infusion [13, 14].

As for this patient, the staged-repair operation was done while the glucose intake and regular insulin infusion were adjusted. The starting dose of the regular insulin infusion was 0.06 units/kg/h, and the rate was increased to 0.1 units/kg/h in the next hour. Insulin discontinuation was based on a recommendation to stop insulin infusion in a neonate if the blood glucose level is less than 200 mg/dL [14]. Frequent blood sugar sampling should be done even after no insulin is infused. Because of poor organ function, the half-life of insulin could be prolonged and result in late hypoglycaemia.In this case, a low-normal blood sugar level was found in the 2nd hour after discontinuation of the intravenous regular insulin.

Insulin availability depends on several factors, such as the tube material, the length of the insulin tubing and the timing of the insulin usage. In this case, insulin-tube priming with 5 IU/ml of regular insulin solution was performed for 20 min both to improve the insulin bioavailability and to reduce insulin absorption by the tubing material. We could not measure the amount of insulin absorption by the insulin tube but hypothesize that insulin absorption via the tubing resulted in a delayed insulin response. Further studies are needed to find an efficient way of handling intravenous insulin infusions, including the proper tube length and size, the insulin volume needed for tube priming, and the time for tube priming [14, 15]. Lowering of the blood sugar could then be achieved effectively by increasing both the dose of the insulin infusion and its bioavailability.

In retrospect, even though gastroschisis is a surgical emergency, a general anaesthesia and operative-theatre procedure may not be necessary on the very 1st day. To avoid intra-operative stress arising from the anaesthesia and surgery in this severe hyperglycaemic-infant, consideration could have been given to performing the silo bedside.

## Conclusion

To date, there are no reports of an association between neonatal hyperglycaemia and gastroschisis. Anaesthesiologists should therefore be aware of the possible, perioperative, dysglycaemic conditions of these patients. The proper timing of surgery and appropriate, pre-anaesthetic preparation are necessary to reduce the morbidity and mortality related to hyperglycaemia and gastroschisis.

## Consent

Written informed consent was obtained from the patient’s legal guardians for publication of this Case Report and any accompanying images.

## Abbreviations

mmol/L: millimole per liter
mg/dL: milligram per deciliter
mg/kg/min: milligram per kilogram per minute
ml/h: milliliter per hour
GIR: glucose infusion rate
POCT: point-of-care testing
ICU: intensive care unit
NICU: neonatal intensive care unit
